# Neuroimaging and Neurocognitive Correlates of Aggression and Violence in Schizophrenia

**DOI:** 10.6064/2012/158646

**Published:** 2012-09-05

**Authors:** Elisabeth M. Weiss

**Affiliations:** Department of Psychology, Karl-Franzens University of Graz, University-Platz 2, 8010 Graz, Austria

## Abstract

Individuals diagnosed with major mental disorders such as schizophrenia are more likely to have engaged in violent behavior than mentally healthy members of the same communities. Although aggressive acts can have numerous causes, research about the underlying neurobiology of violence and aggression in schizophrenia can lead to a better understanding of the heterogeneous nature of that behavior and can assist in developing new treatment strategies. The purpose of this paper is to review the recent literature and discuss some of the neurobiological correlates of aggression and violence. The focus will be on schizophrenia, and the results of neuroimaging and neuropsychological studies that have directly investigated brain functioning and/or structure in aggressive and violent samples will be discussed as well as other domains that might predispose to aggression and violence such as deficits in responding to the emotional expressions of others, impulsivity, and psychopathological symptoms. Finally gender differences regarding aggression and violence are discussed. In this context several methodological and conceptional issues that limited the comparison of these studies will be addressed.

## 1. Introduction

Aggression and violence are declared as leading public health problems and therefore violence in the community has obvious social relevance for political, criminal justice, and health care systems [1]. Epidemiological studies have shown that individuals diagnosed with major mental disorders such as schizophrenia are more likely to be engaged in violent behavior than the general population [2–6]. However, violence committed by persons with schizophrenia is a heterogeneous phenomenon. It is unquestionable that societal influences, such as poverty, discrimination, exposure to violence, and physical abuse, play a key role in the genesis of violence. For example, individuals who are abused as children have increased levels of violence in adulthood [7, 8]. Furthermore, in a recent meta-analysis, Fazel et al. [3] could show that most of the risk for violence appears to be mediated by substance abuse comorbidity. Although aggressive acts can have numerous causes, research about the underlying neurobiology of violence and aggression in schizophrenia can lead to a better understanding of the heterogeneous nature of that behavior and can assist in developing new treatment strategies. In response, a large number of studies have been published to determine the roots of violence and aggression, but the underlying neurobiology is only just beginning to be understood. 

 The purpose of this paper is to review the recent literature and discuss some of the neurobiological correlates of aggression and violence in the literature. The focus will be on schizophrenia and the results of neuroimaging studies and neuropsychological assessments that have directly investigated brain functioning and/or structure in aggressive and violent schizophrenic samples. Additionally, other domains that might predispose to aggression and violence such as deficits in responding to the emotional expressions of others, impulsivity, and psychopathological symptoms will be discussed. In this context, the relevance of various methodological and conceptual issues on the results of these studies will be emphasized. 

## 2. Evidence from Brain Lesions and Brain Imaging Studies

Over the last years, there has been an increasing body of data on neuropsychiatric disorders that raise the question about a possible relationship between the abnormal function of specific regions of the brain and the occurrence of violent and aggressive behavior. The regions of the brain most often linked to aggression and violence are the temporal cortex/limbic system (amygdale, hippocampus, cingulate gyrus, portions of the thalamus, and hypothalamus and their connections) and the orbitofrontal cortex (for a review, see Volavka [9]). 

Since the middle of the nineteenth century, case studies of patients with neurodegenerative disorders or after traumatic brain injury have reported about violent and antisocial behavior, impulsivity, and inability to inhibit responses after damage to the orbitofrontal cortex [10–13]. For example the dramatic case of Phineas Cage, a railroad worker, who had an iron bar driven through the orbito-frontal cortex as a result of an explosion [14]. After the accident he became belligerent, socially inappropriate, unrealistic, and impersistent. The Vietnam Head Injury Study (VHIS) found that subjects with lesions limited to the frontal lobes tended to show about 10% more aggressive and violent behaviors compared with patients with nonfrontal head injury and controls without head injury [15]. Furthermore, persons with frontal network damage acquired before the age of 8 have also been reported to have adult histories of recurrent impulsive, aggressive, and antisocial behavior [16–18]. Moreover, reports have found higher rates of antisocial behavior in patients with frontotemporal dementia, even when compared with equally cognitively impaired patients with Alzheimer's disease [19, 20].

Morphometric neuroimaging studies of aggressive and violent subjects have consistently found frontal lobe abnormalities (for reviews, see [21–25]). However, to date the structural MRI literature produced inconsistent results regarding the volume size abnormalities in aggressive and violent patients, with most studies finding reduced volumes of frontal structures in violent patients (see the meta-analysis of 43 structural and functional imaging studies of Yang and Raine [26]), while others reported increased volumes in these brain regions [27–31]. Furthermore abnormalities in cortical thickness in the ventromedial prefrontal cortex [32] and reduced white matter integrity in the ventral prefrontal cortex, measured with diffusion tension imaging (DTI) [33], were associated with aggressive attitudes and violence. 

Only a handful of studies examined differences in grey matter volumes between violent and nonviolent schizophrenic patients. Yang et al. [34] found reduced gray matter volumes in the hippocampus and parahippocampal gyrus in murderers with schizophrenia, in the parahippocampal gyrus in murderers without schizophrenia, and in the prefrontal cortex in nonviolent schizophrenic patients compared to normal controls. Volume reductions in the hippocampus in violent schizophrenic patients were also seen in other studies [35, 36]. Since hippocampal/parahippocampal deficits have been linked to memory impairments and affective dysregulation, Yang et al. [34] hypothesized that volume reductions in the hippocampus may predispose individuals with schizophrenia to be less sensible to social and emotional signs, which might contribute to the generation of conflicts and the inability to recognize signals for solution, leading to conflict escalation. Less consistent patterns of gray matter volume reductions were found in other studies, comparing violent and nonviolent schizophrenic patients. These studies are reporting about reductions in mesial temporal structures [37], in the amygdale [38], in the sensorimotor cortex [35], in orbitofrontal cortex [36], in the cerebellum, and in the region around the supramarginal gyrus [39]. One study [27] found larger left orbitofrontal cortex grey matter volumes in aggressive schizophrenic patients. These inconsistent results in structural brain volumetric studies can be explained by methodological differences between studies including differences in the study samples (inclusion of multiple diagnostic categories such as schizoaffective patients or patients with comorbidity of personality disorders or psychoactive substance misuse) and treatment characteristics.

Studies focusing on the patterns of brain activation using PET, SPECT, and FMRI have documented focal decreases in frontal and temporal cortical activity associated with various neuropsychiatric disorders (for reviews, see [20–25]). They provide data on both resting brain activity and task-related activity. 

Using positron emission tomography (PET), Wong et al. [40] reported reduced glucose metabolism in the temporal cortex in violent schizophrenic patients, whereas Raine et al. [41–43] found reduced glucose metabolism in the prefrontal cortex and other regions (superior parietal regions, angular gyrus and corpus callosum) in murderers using the continuous performance tasks to elicit frontal activity. Similarly, in a single photon emission computed tomography (SPECT) Spaletta et al. [44] found reduced prefrontal regional cerebral blood flow in aggressive schizophrenic patients during the Wisconsin Card Sorting Task (WCST). However, one has to keep in mind that the sample size in the few PET and SPECT studies showing an abnormal frontotemporal circuitry in aggressive patients is fairly small and the location of abnormal metabolism varied among patients.

Functional imaging studies (fMRI) on aggression in schizophrenia and other mental disorders produced also inconsistent results, with most studies showing an abnormal activation in the temporoventromedial cortex region, but less consistent patterns in other regions [45–52]. Again, comparison between studies is hampered by methodological differences such as different cognitive activations tasks (e.g., visual verbal working memory tasks, affective words, emotional faces), heterogeneous patient populations, and small sample sizes. Only one study examined the resting state functional connectivity in aggressive schizophrenic patients, showing a significant reduction in functional connectivity between the amygdale and ventral prefrontal cortex [53].

In summary the neuroimaging literature on aggression in schizophrenia and other mental disorders implicates dysfunctions in the frontotemporal circuitry (for reviews, see [21–25, 54]). These findings are consistent with the role of medial temporal and orbitofrontal regions in emotional processing and executive cognitive functioning, which encompasses abilities such as attention, planning, organization, abstract reasoning, self-monitoring, and the ability to use feedback to modulate behavior. This has been speculated to lead to cognitive biases that increase the chances of behaving aggressively in response to stressful and provocative situations [4]. The reported abnormalities in prefrontal size or activity may, therefore, represent a predisposition to affective states relevant to aggressive behavior, without necessarily signifying an incapacity to avoid actual violent acts, and no study has reliably demonstrated a characteristic pattern of frontal network dysfunction predictive of violent crime (for review, see Filley et al. [55]). Although the etiological implications of these prefrontal network dysfunctions are not fully understood, there is converging evidence that cognitive deficits may underlie early school failure, dropouts, alcohol and drug use, and ultimately, encounters with the legal system as violent offenders (for review, see [9, 10]). 

## 3. Evidence from Neuropsychological Assessments

### 3.1. Deficits in Executive Functions

It is fairly common that the majority of schizophrenic patients have profound deficits on measures of executive functions. At the descriptive level, patients with schizophrenia seem to have difficulties selecting relevant information from their environment and attaching an appropriate meaning to that information. Green [56] provided the following definition of executive function: 

“Executive functioning refers to a host of neurocognitive activities that are associated with the prefrontal cortex such as planning, problem solving, shifting cognitive set and alternating between two or more tasks.”

Therefore, executive functions allow you to set goals, make and modify mental “models” of actions, organize your activity, focus your attention selectively, and avoid impulses and distractions that could sidetrack you from accomplishing your aims. 

Reviews of the literature tend to support a significant association between prefrontal executive dysfunctions measured by neuropsychological testing and increased antisocial and aggressive behavior [54, 57]. Morgan and Lilienfeld [57] found in their meta-analysis that antisocial groups performed .62 standard deviations worse on executive tests than comparison groups, but the strength of the correlation between deficits in executive functions and antisocial behavior varied according to the types of antisocial behaviors exhibited by study subjects, with criminality and delinquency showing a stronger relationship than conduct disorder, psychopathy, or clinically defined antisocial personality disorder. 

Only a few studies have examined the relationship between performance on neuropsychological tests and violence among schizophrenic patients (see Table 1). However, the results of these studies have been inconsistent. Several studies [58–62] did not find a difference in neuropsychological test performance between schizophrenic patients with aggressive behavior and those without. In contrast, three studies [63–65] found superior performance on neuropsychological measures among violent schizophrenic patients compared to nonviolent patients with schizophrenia. Roy et al. [63] found, in a sample of 20 inpatients with chronic schizophrenia, that violent patients (defined on the basis of chart review and ward behavior) outperformed nonviolent patients on several subscales of the Wechsler Adult Intelligence Scale Revised (WAIS-R): verbal IQ, digit symbol, and block design. A study by Lapierre et al. [64] found a correlation between enhanced performance on both the Wisconsin Card Sorting Test (WCST) and a verbal fluency test and lifetime “number of aggression against another person” among 31 outpatients with schizophrenia. The low-violence group did not achieve significantly better scores than the violent group on any neuropsychological measure in either study. Rasmussen et al. [65] compared 13 violent schizophrenic inpatients with 13 nonaggressive schizophrenic patients and 13 healthy control subjects. Violent schizophrenic patients outperformed nonviolent schizophrenic patients on the Trail Making Test and showed faster reaction time on all reaction time tests but more failed inhibitions on the Go-NoGo test.

Other authors showed that neuropsychological impairments were associated with violence among inpatients with schizophrenia [66–68]. Krakowski et al. [66] classified schizophrenia inpatients into high (*N* = 22), low (*N* = 27), or no violence (*N* = 22) groups on the basis of ward behavior. Patients in the high-violence group were significantly more impaired in the area of integrative sensory and motor function than those in the nonviolent group, as measured by their scores on the Benton Visual Retention Test and the WAIS-R performance IQ, especially on the subtests: digit symbol test and blocking test. On these subtests, violent patients received higher scores in the study by Roy et al. [63]. Adams et al. [67] found that impairment on the Lubria-Nebraska Neuropsychological Battery was related to history of violent arrest but not to inpatient violence in a sample of 37 incarcerated persons with schizophrenia. Barkataki et al. [68] could show that schizophrenic patients with a history of violence produced poorer performance in an executive task (WCST) than nonviolent schizophrenic patients, with the violent patients making significantly more perseverative errors. 

Of the remaining studies none dealt exclusively with schizophrenia. However, in two of the studies a majority of the participants had a diagnosis of schizophrenia. One study compared 23 forensic inpatients, 16 of whom had a diagnosis of schizophrenia, who had committed a violent crime [69]. Impairment on several neuropsychological tests, the judgment of line orientation test, the symbol digit modalities test, the Stroop Interference Test, and the test of nonverbal intelligence, was correlated with the frequency and severity of violent behavior. In a larger study, Krakowski et al. [70] compared 33 inpatients with a history of community violence, as determined by self-report of arrest for violent crime and chart reviews for such arrests, with 69 inpatients who denied arrests for violent crimes. Of the 102 patients included in the study, 72 percent had a diagnosis of schizophrenia or schizoaffective disorder. A history of community violence was significantly related to impairment on some WCST subtests as well as impairment in the finger-tapping test and the Perdue pegboard test (both left handed). Finally, Nestor [71] examined the neuropsychological and clinical correlates of extreme violence retrospectively in young and older inpatients of a forensic psychiatric hospital. The young group exhibited significantly higher rates of learning disabilities and history of childhood conduct disorders, whereas the older group had a significantly higher rate of psychosis. 

Three studies [72–74] aimed to determine models for explaining aggressive behavior in relation to executive functions, psychopathological symptoms, and behavioral variables such as anger. In a study of Song and Min [72] the structural equation model revealed a direct, significant path of the emotion anger to aggressive behavior, whereas schizophrenic symptoms and cognitive functions were indirectly related to aggressive behavior through the relationship between the emotion of anger and aggressive behavior, suggesting that executive dysfunction may affect the stimulant of anger by weakening impulse control. Two other studies [73, 74] could show that executive dysfunction predicted aggressive and violent behavior and psychiatric symptomatology. Serper et al. [73] concluded that patients with executive dysfunctions may not possess the behavioral inhibition skills needed to cope with the presence of symptoms and other stressful events that accompany acute psychosis and hospitalization which may result, consequently, in increased manifestations of aggressive behavior. Finally, a recent study Krakowski and Czobor [75] could show that in schizophrenic patients executive function was a strong predictor of response to atypical antipsychotic medication, with clozapine exerting an antiaggressive effect even in the presence of executive dysfunction. 

To summarize, a large number of studies have found that dysfunctions of the frontal cortex are associated with limitations in the executive capacity to regulate aggressive behavior (for reviews, see [4, 10, 57]). Therefore, neuropsychological deficits can reduce the number of options an individual perceives or has availablity to respond to a given situation. As a component of the human flight and fight response, aggression may serve as an adaptive mechanism. When cognitive dysfunction is present, however, aggression may dominate the behavioral response and override avoidance or withdrawal. Deficits including defective inhibitory control, impulsivity, difficulty in drawing on past learning to recognize a dangerous course of action, impaired capacity to anticipate future consequences of present behavior, and insufficient self-monitoring, may all conspire to conceal more socially desirable courses of action, which may result, consequently, in increased manifestations of aggressive behavior. Although social and environmental factors have major effects on the expression of aggression and violence, neuropsychological integrity also helps to determine the ability to behave in a social acceptable manner. However, studies investigating the relationship between violence in schizophrenic patients and neuropsychological functioning produced mixed results, with reports of better, similar, and worse performance in violent schizophrenic patients. One reason for these inconsistencies in study results may be that the standard tests of executive functions may not detect orbitofrontal or ventromedial prefrontal dysfunction relevant to aggression and criminal behavior. Furthermore, there is a substantial variation in the neuropsychological test batteries used in different studies.

### 3.2. Deficits in Responding to the Emotional Expressions of Others

The expression of emotions and the ability to recognize facial expressions of emotions in other people is an important component of interpersonal communication in humans [75]. Certain facial expressions like happiness, sadness, anger, fear, disgust, and surprise are universally recognized, whereas social emotions such as guilt, shame, arrogance, admiration, and flirtatiousness are particular to cultural and ethnical groups [76, 77]. Emotion is normally regulated in the human brain by a complex circuit consisting of the orbital frontal cortex, amygdala, hypothalamus, anterior cingulate cortex, and several other interconnected regions [78]. Impairments in emotion regulation, emotional inexpressiveness, and other emotion-related deficits are regarded as core features of schizophrenia (for a review, see Borod [78]). Abnormal expression of emotional states usually consists of flattening of affects and inappropriate affect and may precede the onset of psychosis by many years [79]. Abnormal experience of emotion ranges from depression to less frequently manic symptoms. Abnormal recognition is described as impaired ability to recognize facial expression of emotion.

Psychological research underscores especially the relation between deficits in regulation of negative emotions and aggressive and violent behavior [80]. Accurate interpretations of non-verbal cues such as facial expressions are important for normal social interaction [81] and deficient abilities to be appropriately guided by the social cues of others and identify one's emotional state may lead to aggressive and violent behavior [82–85]. Flattening of affect as it is seen in schizophrenia may further contribute to aggressive behavior insofar as a failure to express emotions can lead to a reliance on maladaptive ways of expressing emotions, such as through verbal and physical aggression [86].

Violent offenders show deficits in recognizing negative facial expressions of emotions [87, 88], a deficit associated with many other forms of psychopathology such as autism spectrum disorders, schizophrenia, and obsessive compulsive disorder. More specifically, previous studies could show that aggressive individuals show a negative emotional bias for ambiguous facial expressions [89]. Violent schizophrenic patients were more likely to perceive anger in emotionally neutral faces [90] and showed a poorer ability to discriminate between intensity of emotions [61]. Dodge and McNiel et al. [91, 92] predicted that a cognitive style characterized by hostile attributions increases the risk of violence because in ambiguous situations aggressive individuals who attend to fewer emotional cues are biased towards aggressive cues and display deficits in what is described as affective perspective taking. Furthermore, some authors suggest that mentalizing, which is the ability to attribute mental states to oneself and others, can be viewed as an inhibitor of violent and aggressive behavior [93]. Two studies [94, 95] could show that violent schizophrenic patients have deficient emotional but intact cognitive mentalizing abilities. 

Another line of research is investigating the relationship between personality traits and aggression. The relationship between antisocial behavior or psychopathy and violence is well established (for a review, see Walsh et al. [96]), and a meta-analysis could show functional impairments in orbitofrontal, dorsolateral frontal, and anterior cingulate cortex in antisocial behavior [97]. Among schizophrenic patients antisocial personality disorder and psychopathy are associated with early onset and persistent violent offending [98, 99].

Bettencourt et al. [100] conducted a comprehensive review to understand the relationship between personality and aggressive behavior, under provoking and nonprovoking conditions. The authors could show that trait aggressiveness and trait irritability influenced aggressive behavior under both provoking and neutral conditions but that other personality variables (e.g., trait anger, type A personality, dissipation rumination) influenced aggressive behavior only under provoking conditions. Since aggression is closely linked to hypervigilance towards stimuli that could be perceived as threatening [101, 102] one other important symptom, possibly related to outbreaks of aggression and violence, particularly in adolescents and young adults, may be “gelotophobia” (from gelos, Greek for laughter), which is the fear to being laughed at [103, 104]. Similar to violent individuals persons with higher levels of gelotophobia have deficits in downregulating their negative affect, show a greater anger proneness, and have a tendency to recall interpersonal situations with a higher intensity of negative feelings [105–107]. The suspicion that gelotophobia may be related to aggression and violence had recently been fuelled by anecdotal evidence suggesting that perpetrators of violent acts such as school shootings had a horror of being mocked and may have taken revenge for having been laughed at [108, 109]. However, more research is needed to extrapolate this relationship between gelotophobia or other personality factors and aggressive behavior to schizophrenic patients. 

## 4. Association between Impulsivity and Violence

A further factor mediating the brain-violence relationship which is also linked to the frontal lobe is impulsivity, a complex, multifaceted construct including cognitive, personality, and behavioral components (e.g., sensation seeking, risk taking, and self control) [110]. In general aggression can be distinguished into a goal-directed predatory/premeditated aggression and a reactive/impulsive aggression [111]. Premeditated aggression occurs without provocation and is defined as a planned, conscious, and controlled aggressive act that is instrumental in nature. Impulsive aggression is more reactive and typically described as an emotionally charged aggressive response either unprovoked or out of proportion to the provocation leading to agitation and a loss of behavioral control. Since impulsive individuals have difficulties inhibiting their urges this behavioral style may result in risky behavior, including drug and alcohol abuse [112]. Alcoholintoxication for example, reduces behavioral inhibition and has been linked with aggression in a number of studies [113–116]. Prior research has shown that poor impulse control is associated with aggression and violence in men but not women [117–120]. Dysfunctions of the ventromedial prefrontal region (VMPFC), including the orbitofrontal cortex (OFC), anterior cingulate cortex (ACC), and medial prefrontal cortex, as well as the amygdala were associated with impulsive-aggressive behavior [33, 121–125], whereas individuals with predominantly premeditated aggression showed no prefrontal deficts [125]. Additionally, neuropsychological studies have shown a clear link between impulsive-aggressive behavior and problems in executive functioning [e.g., [126–128]]. Again, some studies could show that individuals who present with marked premediated aggression such as psychopaths do not present with poor performance on general measures of frontal-lobe functioning [129, 130]. Accumulating evidence points towards an important role of several neurotransmitters such as serotonin, dopamine, and noradrenalin in impulsive behavior, and recent findings also implicate glutamate and cannabinoid neurotransmission in impulsivity (for a review, see Pattij and Vanderschuren [131]). Therefore, impulsive behavior may be responsive to pharmacological treatment, and several studies could show that agents with serotonergic-enhancing properties such as serotonin-reuptake inhibitors [132–134] as well as antiepileptic dugs may reduce incidences of impulsive aggression, but not premediated aggression (for reviews, see Huband et al. [135] and Stanford et al. [136]). 

### 4.1. Association between Violence and Psychopathological Symptoms

To some extent the increased risk of violence in patients with schizophrenia may be related to specific psychotic symptoms, such as delusions of thought insertion, thought control, and persecution or to command hallucinations, but the link between violence and psychotic symptoms can be minimal. 

Link and Stueve [137] reported that violent behavior was associated with the presence of “threat/control-override symptoms”: delusions of domination by external force, thought insertion, and feeling that people wish to harm the patient. However, epidemiological study did not investigate whether there was a temporal linkage between delusions and violence. Junginger et al. [138] concluded that delusional motivation of violence appeared to be rare, though 40% of the violent subjects in that study reported that they had committed at least one serious act of violence that was definitely motivated by delusions. Taylor [139] interviewed 121 incarcerated forensic psychotic (mostly schizophrenic) patients about the motives for the offenses they had committed while living in the community. The author estimated that 82% of their offenses (violent and nonviolent offenses combined) were probably attributable to their illness and that psychotic symptoms probably accounted directly for most of the very violent behavior, but considerable time elapsed between the offenses and the interviews, and the respondent may have had reason to represent their criminal behavior as driven by mental illness.

Some patients may commit violent acts in response to auditory command hallucinations. In a sample of 93 psychiatric inpatients, Junginger [140] rated the level of dangerousness of the most recently reported command hallucinations and the degree to which the patient complied with the command. He concluded that psychiatric patients who experience command hallucinations are at risk for dangerous behavior. However, others have concluded that although experienced by many patients with schizophrenia and often violent in content, command hallucinations may not be important predictors of violence [141, 142]. In a comparison of violent and nonviolent patients with schizophrenia (*n* = 31 per group) Cheung et al. [143] reported no association between command hallucinations and violence; yet, violent patients were more likely to have had persecutory delusions and were more severely psychotic than the nonviolent patients. 

In summary, there is still a controversy regarding the relationship between psychotic symptoms in schizophrenic patients and aggressive behavior with some authors reporting that positive psychotic symptoms such as delusions of thought insertion, thought control and persecution, or command hallucinations were associated with a history of assaults [144, 145], whereas others could not observe association between positive symptoms of schizophrenia and violence [62]. 

### 4.2. Gender and Aggression

In healthy people, aggression is arguably the most salient emotional behavior differentiating the sexes with men showing greater abundance [146], including arrests for homicide and violent crimes [147]. Studies of aggression in psychiatric disorders showed variable findings (for a review, see Maccoby and Jacklin [146]). Several studies have suggested that psychiatric disorders, especially schizophrenia, reduce the gender difference and in hospitalized psychiatric patients eliminate it altogether [148–151]. However, in order to understand better gender differences in violence, multiple manifestations of violence must be differentiated and the complexity of the phenomenon must be taken into consideration. For example, Hiday et al. [152] demonstrated that male subjects had a greater prevalence of more serious violence involving weapons or injury in the 4 months preceding hospitalization, but there were no gender differences when measures of violence that were more inclusive (incorporating threats as well as fights not involving weapons or injuries) were used. Additionally, Weiss et al. [153] could show that physical aggression and antisocial behavior in the community were more pronounced in schizophrenic men compared to schizophrenic women. In a study by Krakowski and Czobor [154], women with major psychiatric disorders were more verbally abusive and threatening during their hospitalization than the men. This finding is consistent with the predominance of verbal aggression reported in women showing that in the general population, women throughout life are more likely to use verbal than physical aggression [155]. A meta-analytic review of studies on gender differences in the general population [156] also indicated that the male overrepresentation in aggression is more distinct for severe physical aggression.

## 5. Conclusions

Violent and criminal behavior in schizophrenic patients is caused by multiple, probably interacting causal factors. Dysfunctions in the frontotemporal circuitry appear to be the most consistent feature in aggressive and violent schizophrenic patients. Limitations in the executive capacity and/or deficits in the interpretation of emotional signals such as facial affects can lead to cognitive biases that increase the chances of behaving aggressively in response to stressful and provocative situations. However, studies elucidating the relationship between neuroimaging results, neurocognitive functioning, personality traits, and antisocial and violent behavior produced inconsistent results. Most studies investigating the neurobiological correlates of aggressive and violent behavior are limited by several methodological problems. Notably, the definition and measurement of violence varied between studies. Most studies focused on community violence, while only few studies examined inpatient violence, which is not necessarily predicted by previous community violence [157]. Additionally, the number of aggressive behavior and violent incidents was assessed differently between the studies reviewed in this paper, with some authors using patients self-reports, while others used psychiatric files, reports by ward staff, or official records of arrests and convictions. Furthermore, inconsistent findings can result from sample heterogeneities such as comorbidity with antisocial personality disorder/psychopathy or substance abuse. Over and above, differences in the medication type and dosage can add to the variance in study results. Atypical antipsychotic medication has shown greater promise in treating neuropsychological deficits than conventional antipsychotic drugs [158]. In addition, atypical antipsychotic medication, particularly clozapine, has been shown to reduce aggressive and violent behavior in schizophrenic patients [75, 159–162]. The failure to take account of type and dosage of antipsychotic medication in some studies may have limited the detection of group differences in neurocognitive performance and aggressive behavior. Further methodological problems in the literature include a lack of prospective data, small samples sizes, nonrepresentative samples for the criminal population, a lack of adequate controls for known violent risk factors such as drug and alcohol misuse, physical and sexual abuse, family break down, and poverty. Future studies are necessary that examine the course of cognitive deficits, psychopathological symptoms, and aggressive behavior in schizophrenic patients at various stages of patient illness. Since several studies found an association between some genes such as the catechol-O-methyltransferase (COMT) and aggression and violence (for review, see Soyka [54]) the combination of neuroimaging, neuropsychological, and genetic studies might broaden our understanding of the neurobiological basis that underlies aggressive and violent behavior. This interdisciplinary research may lead to an earlier identification of likely aggressors and may result in the formation of individualized treatment plans, which can reduce patients' symptoms and the propensity for criminal behavior and aggression.

## Figures and Tables

**Table 1:** Results of relevant neuropsychological studies in aggressive schizophrenic patients.

Authors	Sample characteristics	Results of relevant neuropsychological studies
Lafayette et al. [58]	96 schizophrenic patients (34 violent arrests, 23 nonviolent arrest, 39 no arrest)	No significant differences between the groups in the WAIS, Wisconsin Card Sortig Test, Trail Making Test, Verbal fluency Test, Stroop Test, American National Adult Reading Test

Krakowski et al. [59]	32 transiently violent schizophrenic patients, 27 persistently violent schizophrenic patients	No difference in the average performance and verbal IQ on the WAIS-R

Wong et al. [60]	20 male schizophrenic patients who had committed several violent offenses, 19 male schizophrenic patients who had committed only 1 violent offense.	No difference in the WAIS performance between the groups

Silver et al. [61]	35 violent patients with chronic schizophrenia or schizoaffective disorder, 35 nonviolent schizophrenic patients, 46 healthy controls	No difference between violent and nonviolent schizophrenic patients in the cognitive test battery

Fullam and Dolan [62]	33 violent and 49 nonviolent male forensic inpatients with schizophrenia	No difference between violent and nonviolent schizophrenic patients in the cognitive test battery but lower IQ scores in the violent schizophrenic patients

Roy et al. [63]	20 chronic, treatment-resistant inpatients with schizophrenia; (11 violent and 9 low-violent men)	Violent patients outperformed nonviolent patients on the verbal IQ, digit symbol test, block design

Lapierre et al. [64]	31 schizophrenic men (outpatients) and 30 healthy control subjects	Higher level of violence in the more cognitively functional individuals on the Wisconsin Card Sorting Test and Controlled Oral Word Association test

Rasmussen [65]	13 inpatients aggressive schizophrenic patients from a maximum security psychiatric unit, 13 nonaggressive schizophrenic patients, 13 healthy controls	Violent schizophrenic patients outperformed nonviolent schizophrenic patients on the Trail Making Test and showed faster reaction time on all reaction time tests but more failed inhibitions on the Go-NoGo test

Krakowski et al. [66]	28 high, 27 low, and 34 nonviolent schizophrenic patients	High-violent patients were more impaired on the Benton Visual Retention Test, digit symbol test, block design and had lower performance IQ scores on the WAIS-R

Adams et al. [67]	37 male schizophrenic patients	History of violent arrests but not inpatients violence was associated with impairment on the Luria Nebraska neuropsychological battery

Barkataki et al. [68]	13 individuals with a history of serious violence and a diagnosis of antisocial personality disorder, 13 individuals with a history of violence and schizophrenia, 15 individuals with schizophrenia without a history of violent behaviour, 15 healthy control subjects	Violent schizophrenic patients had a higher number of errors in the Wisconsin Card Sorting Test

Foster et al. [69]	1 year prospective study of aggression in 23 male forensic psychiatric inpatients	Scores on Stroop Color Word Tasks, Judgment of Line Orientation Test, Symbol Digit Modalities Test only significantly predicted frequency but not severity

Krakowski et al. [70]	33 psychiatric violent inpatients versus 69 nonviolent psychiatric inpatients	History of community violence was related to impairment on some Wisconsin Card Sorting Subtests, finger tapping, Perdue pegboard

WAIS-R: Wechsler Adult Intelligence Scale-revised.
